# Neighbourhood flower diversity increases reproductive success of *Lantana hypoleuca* Briq (Verbenaceae)

**DOI:** 10.1111/plb.70044

**Published:** 2025-05-23

**Authors:** M. D. de Amorim, J. P. R. Borges, A. C. P. Machado, A. R. Rech, P. K. Maruyama, P. J. Bergamo

**Affiliations:** ^1^ Programa de Pós‐Graduação em Ecologia, Conservação e Manejo da Vida Silvestre, Instituto de Ciências Biológicas Universidade Federal de Minas Gerais Belo Horizonte Minas Gerais Brazil; ^2^ Programa de Pós Graduação em Ciência Florestal Universidade Federal dos Vales do Jequitinhonha e Mucuri Diamantina Minas Gerais Brazil; ^3^ Faculdade Interdisciplinar de Humanidades Universidade Federal dos Vales do Jequitinhonha e Mucuri Diamantina Minas Gerais Brazil; ^4^ Centro de Síntese Ecológica e Conservação, Departamento de Genética, Ecologia e Evolução, Instituto de Ciências Biológicas Universidade Federal de Minas Gerais Belo Horizonte Minas Gerais Brazil; ^5^ Departamento de Biodiversidade, Instituto de Biociências Universidade Estadual Paulista, campus Rio Claro Rio Claro São Paulo Brazil

**Keywords:** Cerrado, facilitation, floral diversity, heterospecific flowers, plant–pollinator interactions, pollination community, pollinator sharing

## Abstract

- The attraction of floral visitors depends on intrinsic plant traits and the surrounding floral abundance and diversity. Therefore, it is important to consider the conspecific and heterospecific co‐flowering context to understand plant–pollinator interactions and, consequently, plant reproductive success. We investigated the influence of the floral neighbourhood on pollination of *Lantana hypoleuca* (Verbenaceae) in the Brazilian Campos rupestres.
- We evaluated how flower richness, conspecific and heterospecific abundance, and similarity in corolla length influence reproductive success (conspecific pollen deposition, heterospecific pollen deposition, and likelihood of fruit production) of *L. hypoleuca* individuals. For this, we evaluated the surrounding neighbourhood of 20 *L. hypoleuca* individuals.
- We recorded 73 visits of bees, butterflies, and wasps. Conspecific pollen was not related to any of the studied variables, while heterospecific pollen increased with plant richness. Finally, the likelihood of fruit production increased with plant species richness and decreased with conspecific floral abundance.
- The positive effect of plant species richness on plant reproduction suggests facilitative interactions, in which higher community diversity may attract a more diverse set of pollinators to *L. hypoleuca*. Our results stress the importance of assessing multiple community features and plant reproductive steps to better understand the complex co‐flowering effects on pollination and reproduction.

## Graphical abstract

A neighbourhood plant richness increases reproductive success.

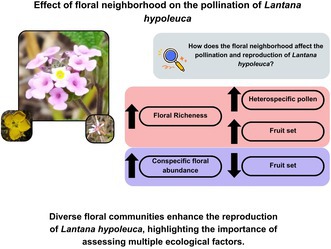

## INTRODUCTION

About ~90% of Angiosperms depend on animal pollination (Ollerton *et al*. 2011; Tong *et al*. 2023), and many floral traits, such as colour and shape, are essential for attracting pollinators (Dudash *et al*. 2011). In addition to flower traits, attraction of floral visitors also depends on individual floral display and the abundance and diversity of other plants in the neighbourhood (Shibata & Kudo 2020; Sritongchuay *et al*. 2021). This is because plants often share generalist pollinators with other plant species (Waser *et al*. 1996; Resasco *et al*. 2021). For instance, plant communities composed of species with high floral trait similarity are often interpreted as the result of pollination facilitation (Sargent & Ackerly 2008). Therefore, it is important to consider the conspecific and heterospecific co‐flowering context to understand pollination patterns, even for a single plant species (Bergamo *et al*. 2018; Sritongchuay *et al*. 2021).

Heterospecific neighbours can influence multiple aspects of plant reproduction, including the abundance and diversity of pollinators visiting an individual plant (Lázaro *et al*. 2009), pollen deposition (Bergamo *et al*. 2020), and seed production (Nottebrock *et al*. 2017). Co‐flowering plant neighbours may attract greater pollinator diversity than monospecific plant neighbourhoods (Lázaro *et al*. 2009), especially if species exhibit distinct floral traits (Ghazoul 2006; Fornoff *et al*. 2017). On the other hand, trait similarity between plant species may attract a variety of pollinators without necessarily resulting in more visits to each plant (Fornoff *et al*. 2017). Plants with similar traits, such as corolla length and opening, flower size, and floral reflectance, may also engage in facilitative interactions because trait similarity facilitates pollinator sharing (Bergamo *et al*. 2020; Lopes *et al*. 2021). In this context, a community rich in species with similar morphologies may attract more pollinator visits (Carvalheiro *et al*. 2014; Bergamo *et al*. 2020) but will also receive more heterospecific pollen (Arceo‐Gómez & Ashman 2014). Nevertheless, much remains unclear regarding how conspecific and heterospecific flowers in a neighbourhood mediate distinct consequences of plant–pollinator interactions, such as pollen deposition and fecundity.

Facilitative interactions lead to enhanced pollinator visitation between plant species sharing pollinators, ultimately increasing plant reproductive success (Ghazoul 2006; Tur *et al*. 2016; Bergamo *et al*. 2024). On the other hand, competition occurs when sharing pollinators causes heterospecific pollen deposition (Arceo‐Gómez & Ashman 2014) and per‐plant reduction of floral visitation rates, leading to reduced reproductive success (Mitchell *et al*. 2009; Ha *et al*. 2020; Johnson *et al*. 2022). In the competition context, reduced levels of pollination niche overlap are important to avoid the negative consequences of pollinator sharing (Wei *et al*. 2021). Nevertheless, heterospecific pollen deposition does not always have strong negative effects (Streher *et al*. 2020; Lopes *et al*. 2021). Facilitation and competition often occur simultaneously in plant communities, and the interaction sign depends on the plant species, community context (i.e., plant neighbourhood), and scale (Lázaro *et al*. 2015; Bergamo *et al*. 2020; Ye *et al*. 2023). Given this complexity, evaluating multiple aspects of the plant neighbourhood, such as abundance, richness, and flower morphology, is important to untangle facilitative vs. competitive interactions for pollination.

We investigated the reproductive biology of *Lantana hypoleuca* Briq. in the Brazilian Campos rupestres. We explored how similarity in corolla length, plant richness, and heterospecific and conspecific neighbour abundance affect pollen deposition and fruit set. In facilitative interactions, we hypothesize that individuals of *L. hypoleuca* in plots with more similar corolla length compared to other plant species will share more pollinators, resulting in more heterospecific and conspecific pollen deposition, and this could result in higher reproductive success. Moreover, we expected a positive effect of richness and floral abundance of heterospecific plants on conspecific and heterospecific pollen deposition, and reproductive success. Therefore, we expected a low negative effect of heterospecific pollen deposition (Streher *et al*. 2020; Lopes *et al*. 2021). On the other hand, if plants in the neighbourhood are competing for pollinators, we expect that floral similarity between the plants will lead to greater deposition of heterospecific pollen and a larger reduction in reproductive success. In contrast, in plots where *Lantana* is dominant, it will receive more conspecific pollen and less heterospecific pollen, leading to higher reproductive success.

## MATERIAL AND METHODS

### Study area

This study was carried out in an area of 240 ha of Campos rupestres at the campus Juscelino Kubitschek of the Universidade Federal dos Vales do Jequitinhonha e Mucuri, Diamantina, Minas Gerais, Brazil (18°11′48.23″S, 43°34′8.74″W). *Campos Rupestres* is a kind of mountain grassland vegetation and exhibits remarkable plant diversity and endemism. Despite covering <1% of Brazil's territory, it accounts for over 10% of its plant diversity (Silveira *et al*. 2016). The study site is in the central region of the Serra do Espinhaço Mountain range, at an elevation of ca. 1300 m a.s.l. It is characterized by rocky outcrops and shallow soils. The climate is mesothermal temperate (Cwb type). Sampling was conducted between October and December 2021, coinciding with the onset of the rainy season in this region, when more species bloom.

### Study species

**Fig. 1:**
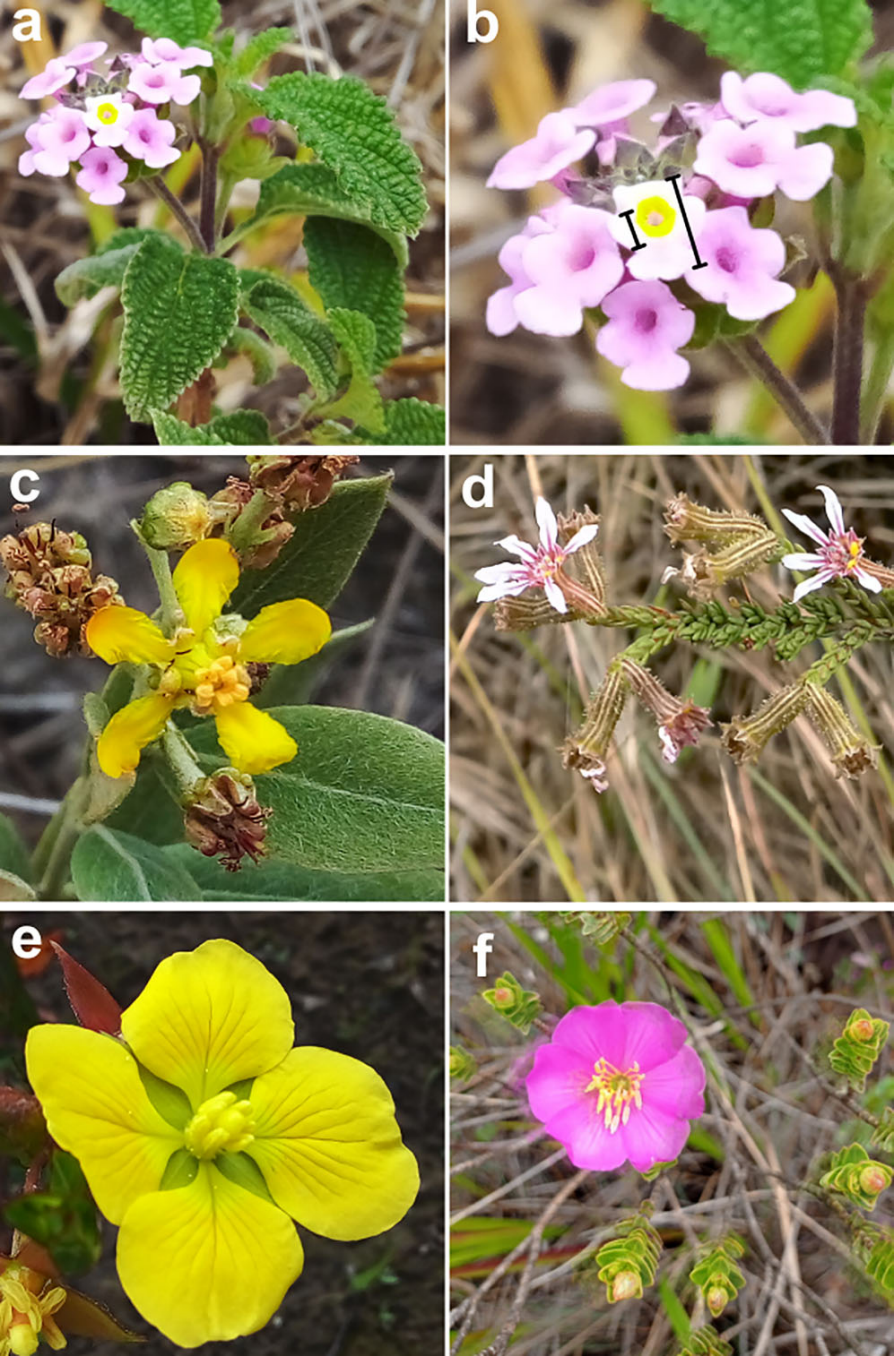
*Lantana hypoleuca* (Verbenaceae): (a) inflorescence, (b) detail of a flower, showing central yellow guide. (c)–(f) Floral diversity found during the study on the Campos Rupestres in Diamantina, MG, Brazil. (c) *Tetrapterys microphylla* (Malpighiaceae); (d) *Cuphea diosmifolia* (Lythraceae); (e) *Ludwigia myrtifolia* (Onagraceae); (f) *Lavoisiera sampaioana* (Melastomataceae).

*Lantana hypoleuca* is a shrub, typically between 0.5 and 2.0 m high. It has lilac flowers with a yellow nectar guide and vinaceous ripe fruits (Fig. 1; Silva 2020). The species is native to Brazil and occurs in the Caatinga, Cerrado, and Atlantic Forest biomes (Silva 2020). In addition, *L. hypoleuca* is visited by generalist pollinators, such as bees, butterflies, and hummingbirds, and is also capable of self‐pollination (Queiroz 2018; Lopes *et al*. 2021; Amorim *et al*. 2022).

### Sampling design

We marked 20 individuals of *L. hypoleuca* and delimited a circular plot of 1.5 m radius around each individual. These individuals were selected based on the criteria of being in bloom, located at a minimum distance of 10 m from each other, and considering the heterogeneity of rupestrian grassland landscapes, such as rocky outcrops and open field areas. In hyperdiverse plant communities, such as Campos rupestres, plant species form small populations restricted to small areas (Silveira *et al*. 2016). Therefore, we sampled in an area with a higher abundance of individuals relatively close to each other to establish the plots. In each plot, we recorded the floral visitors to *L. hypoleuca* through focal observations for 1 h per individual (except P01, P02, P10 and P16, which stopped flowering before all observations; see Table S1). The observations were divided into 20‐min sessions on three different days for a total of 19 h of observation. During the observations, we counted the number of flower visitors on the focal plant (Table S1). Before starting the observations, we counted plant species richness within the plots and the number of flowers in each focal *L. hypoleuca* (floral display). During the observations, we also recorded the number of flowers of all *L. hypoleuca* individuals within the plot (conspecific floral abundance) and the sum of the number of flowers of all other species within the plot (heterospecific floral abundance). We considered the conspecific and heterospecific floral abundance as the average of the sum of flowers across all the days of field observation (Table S2). Similarly, we considered plant species richness as the average number of species flowering across all the days of field observation.

### Morphological similarity

To evaluate similarity in floral morphology at the community scale, we measured the corolla length of one flower from a minimum of five individuals of each plant species found within the plot, including *L. hypoleuca*. To measure corolla length, we used individuals of the species found outside the plot when necessary. Corolla length was measured as the distance from the base to the tip of the corolla. Out of the 29 plant species in the plots, we measured the corolla length of 24 species (Table 1). We chose corolla length to represent flower size, which is an important factor for attracting floral visitors. However in a tubular flower such as *L. hypoleuca*, corolla length should also be related to restrictiveness (distance to nectaries). We used these morphological data to calculate the distance between the corolla length of heterospecific flowers and *L. hypoleuca*. Specifically, we subtracted the corolla length of each flower species in the plot from that of *L. hypoleuca*. The similarity in morphology was defined as the mean distance from *L. hypoleuca* to each other species within the plot.

**Table 1:** Plant species found during the study. Code represents the species in Table S3.

	code	corolla length		size
		SD	average	
*Lantana hypoleuca*	Lan_hy	0.27	1.19	20
Asteraceae sp. 01	Ast_01	0.07	0.30	6
*Lepidaploa* sp.	Ast_03	0.66	4.85	10
Asteraceae sp. 02	Ast_04	NA	NA	0
*Jacaranda oxyphylla*	Big_01	0.75	4.46	10
*Chamaecrista choriophylla*	Cha_01	0.21	1.88	4
*Chamaecrista* sp. 01	Cha_02	0.17	1.35	10
*Cuphea diosmifolia*	Cup_01	0.11	0.38	10
*Cuphea disperma*	Cup_02	0.10	0.23	7
*Evolvulus glomeratus*	Esp_01	0.15	0.69	10
N.I 03	Esp_04	0.04	0.10	10
N.I 04	Esp_07	NA	NA	0
N.I 01	Esp_13	0.07	0.19	10
*Pfaffia aphylla*	Esp_16	0.15	0.52	10
N.I 02	Esp_19	0.22	0.70	10
*Borreria verticillata*	Esp_21	1.22	3.70	5
*Marsypianthes chamaedrys*	Esp_25	0.08	1.03	10
*Ludwigia myrtifolia*	Esp_26	0.20	1.25	10
N.I 05	Esp_28	NA	NA	0
N.I 06	Esp_29	NA	NA	0
N.I 07	Esp_30	NA	NA	0
Fabaceae sp. 01	Fab_01	0.19	0.70	10
*Tetrapterys microphylla*	Mal_01	0.05	0.35	10
Malpighiaceae sp. 01	Mal_02	0.15	0.58	10
*Pleroma candolleanum*	Mel_01	0.61	2.87	10
Melastomataceae sp.	Mel_02	0.11	0.53	10
*Lavoisiera sampaioana*	Mel_05	0.23	2.18	10
*Piptolepsis imbricata*	Pip_im	0.12	0.81	10
Velloziaceae sp. 01	Vel_01	NA	NA	0

Corolla length was measured in mm, and size is the number of measured flowers.

### Pollen deposition and reproductive success

We estimated pollen deposition and fruit set of each focal *L. hypoleuca* plant. For pollen deposition, we selected two flowers near senescence from each focal *L. hypoleuca* plant (n = 40). The stigmas of these selected flowers were removed and taken to the laboratory, where they were placed on glass slides with carmine glycerin gelatin to stain the pollen then observed under a light microscope. We counted the number of conspecific and heterospecific pollen grains. To distinguish between conspecific and heterospecific pollen, we used pollen from the anthers of *L. hypoleuca* as a reference. For this, we collected some anthers of *L. hypoleuca* and prepared slides using the same gelatin. We then determined conspecific and heterospecific pollen by comparing the pollen found in the anthers with the pollen observed on the stigma slides. We estimated the average heterospecific and conspecific pollen across the stigmas of each focal individual (Table S3). To assess fruit set, we marked one inflorescence with a minimum of eight buds and a maximum of 51 buds in each focal *L. hypoleuca* individual (Table S3). The reproductive success was the rate between the number of fruits and the total number of marked flowers.

### Pollinator dependence

To estimate pollinator dependence on *L. hypoleuca*, we marked flowers at the bud stage for spontaneous self‐pollination and natural pollination treatments (n = 20 and 23, respectively). In the spontaneous self‐pollination treatment, we bagged buds to prevent visits by pollinators, while the flowers in the natural pollination treatment were left exposed to visitors.

### Statistical analyses

We made three global models to investigate the relationship between *L. hypoleuca* heterospecific and conspecific pollen deposition, likelihood of fruit production, and the plant neighbourhood:

1. The effect of plant neighbourhood (similarity in corolla length, plant species richness, conspecific and heterospecific floral abundance) on heterospecific pollen. Since heterospecific floral abundance and floral richness are correlated, we only used floral richness in the models (cor = 0.57; *t* = 2.99; df = 18; *p*‐value <0.01).
2. The effect of plant neighbourhood (similarity in corolla length, plant species richness and conspecific floral abundance) on conspecific pollen.
3. The effect of plant neighbourhood and conspecific and heterospecific pollen on the likelihood of fruit production. As heterospecific pollen deposition and conspecific pollen deposition are correlated, we only used conspecific pollen deposition in the models (cor = 0.68; *t* = 3.93; df = 18; *p*‐value <0.01).

Heterospecific pollen deposition was log‐transformed in all models to improve model fit, and Gaussian distribution was used for models (1) and (2), while binomial distribution was used for model (3). We fitted generalized linear mixed models (GLMM) with the *glmmTMB* function in the “glmmTMB” package (Brooks *et al*. 2017), the *simulateResiduals* function from the “DHARMa” package (Hartig 2022) and *check_collinearity* function from the “performance” package (Lüdecke *et al*. 2021) to check model assumptions.

As we had many predictors, we performed model selection to avoid overfitting the models. For this, we used the “MuMIn” package (Barton 2024) to identify the most parsimonious models based on Akaike's Information Criterion for small samples (AICc). All data used for analysis are available in Table S4.

Finally, to estimate pollinator dependence of *L. hypoleuca*, we used a Chi‐square test with seed presence or absence in the marked buds as the response variable and treatments (natural and self‐pollination) as predictors.

All analyses were conducted using R software (R Core Team 2023).

## RESULTS

During the observations, we recorded a total of 73 visits (56 by bees, 15 by butterflies, and 2 by wasps; Table S1). The bees visited only flowers of *L. hypoleuca* with the yellow guide, despite some initially approaching flowers without the visible guide.

**Fig. 2:**
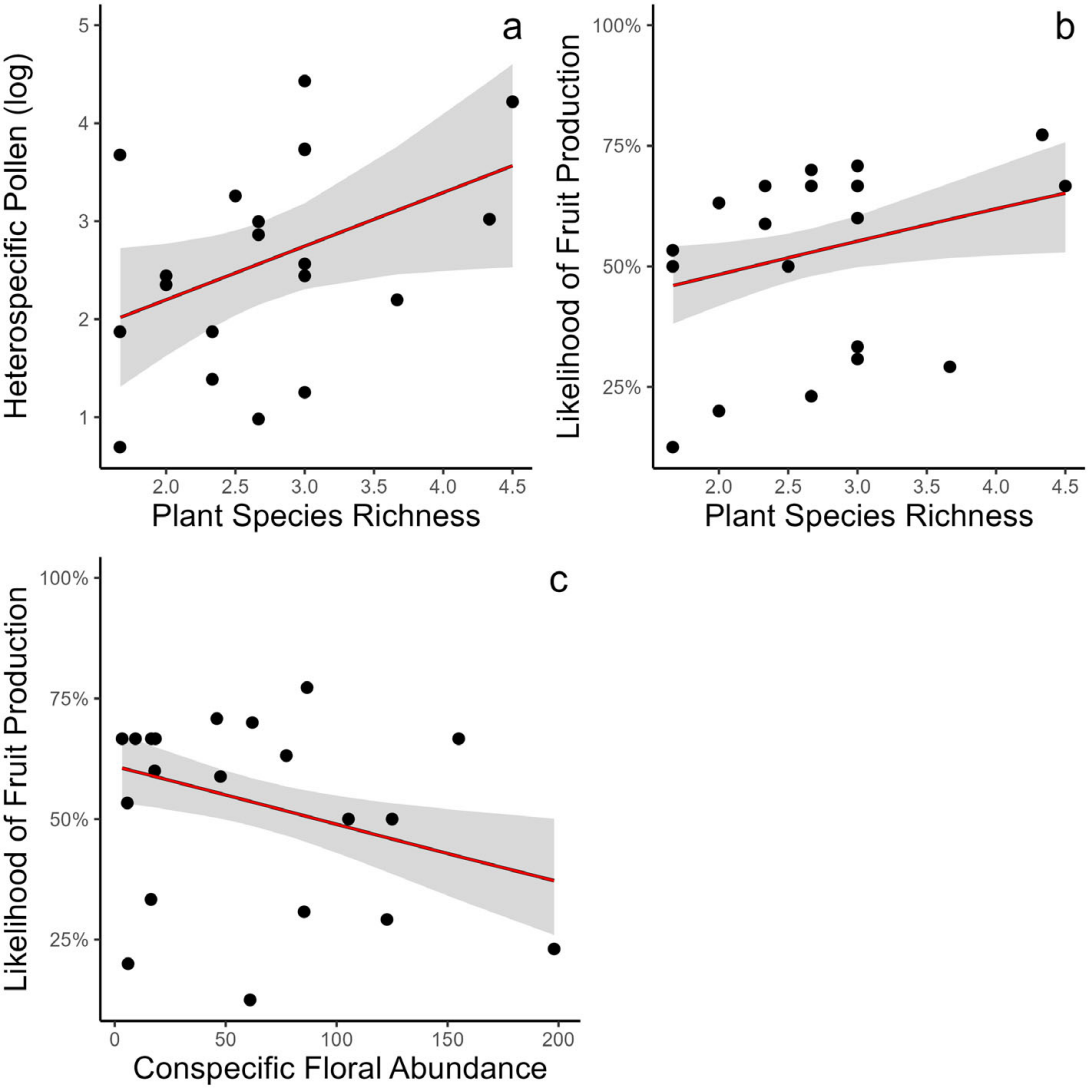
(a) Relationship between heterospecific pollen deposition and plant species richness. Relationships between likelihood of fruit production and (b) plant species richness and (c) conspecific floral abundance. Each dot represents a distinct *L. hypoleuca* individual. Red line indicates significant relationship at *p* < 0.05, grey area represents 95% confidence interval.

Although they were correlated (*r* = 0.68), conspecific and heterospecific pollen deposition did not exhibit the same relationships with the predictor variables. The best model for heterospecific pollen deposition included only plant species richness (estimate = 0.54; *X* ^*2*^ = 3.97; *p*‐value = 0.04; Table 2, Fig. 2a), while only the intercept (null model) was the best model to explain conspecific pollen deposition (Table 2). The likelihood of fruit production increased with plant species richness (estimate = 0.27; *X* ^*2*^ = 4.47; *p*‐value <0.01; Table 2, Fig. 2b) and decreased with conspecific floral abundance (estimate = −0.004; *X* ^*2*^ = 6.92; *p*‐value = 0.03; Table 2, Fig. 2c).

**Table 2:** Model selection results for: heterospecific pollen deposition, conspecific pollen deposition, and fruit set.

response	predictors (models)	AIC	ΔAIC
Heterospecific pollen	~Richness	**62**	**0.00**
	~CFA + richness	62.8	0.81
	~1	62.8	0.83
	~ CFA	64.1	2.06
Conspecific pollen	~1	**204.3**	**0.00**
	~CFA	205.8	1.53
	~Richness	206.2	1.87
	~Corolla length similarity	207.7	2.73
Fruit/buds	~CFA + richness	**112.7**	**0.00**
	~CFA + richness + conspecific pollen	114.2	1.43
	~CFA	114.5	1.78
	~Conspecific pollen	114.7	1.95
	~Richness + conspecific pollen	114.8	2.12

The table shows the best models according to *AICc* until the first *ΔAICc* >2. Richness refers to plant species richness. CFA is conspecific floral abundance. Corolla length similarity is average similarity in corolla length of *L. hypoleuca* in relation to heterospecific plant species. Bold represents the best model.

*Lantana hypoleuca* did not exclusively require pollinators to set fruit, with a fruit set of spontaneous self‐pollination of 0.30 ± 0.47 fruit/flowers. However, natural pollination gave a fruit set of 0.73 ± 0.44 fruits/flowers. Therefore, pollinators enhanced fruit set (*X* ^*2*^ = 6.62; *p* = 0.01).

## DISCUSSION

The plant neighbourhood influenced each step of the reproductive process, from pollen deposition to fruit production. The increase in heterospecific pollen deposition with surrounding plant species richness indicates that pollinator sharing mediates patterns of pollen deposition. Individuals of *L. hypoleuca* increased their reproductive success when surrounded by plants of different species, indicating a facilitative relationship between heterospecific neighbourhood plants and *L. hypoleuca*. Despite not requiring pollinators for reproduction, flowers exposed to visitors showed higher reproductive success, reinforcing the importance of heterospecific neighbourhood plants for the reproductive success of *L. hypoleuca*.

The increase in heterospecific pollen deposition with plant richness indicates that *L. hypoleuca* is receiving visitors from subsequent visits to other plant species. As we hypothesized, the plant species that received more heterospecific pollen also received more conspecific pollen deposition. However, despite the correlation, conspecific pollen deposition did not respond to the neighbourhood in the same way as heterospecific pollen deposition. In addition, reproductive success also increased with plant species richness, suggesting that flowering in diverse plant neighbourhoods is advantageous for *L. hypoleuca*. Previous studies have shown a negative association between heterospecific pollen deposition and plant reproduction (Morales & Traveset 2008; Ashman *et al*. 2020). For *L. hypoleuca*, self‐pollination and the low number of ovules may mitigate the negative effect of heterospecific pollen, as it does not require large amounts of conspecific pollen for reproduction. Even in the presence of heterospecific pollen, a few conspecific grains would be sufficient for fruit set. Especially considering that higher plant species diversity surrounding the focal species can enhance pollinator attraction, increasing heterospecific pollen deposition and fruit set through a facilitation process (Arroyo‐Correa *et al*. 2024; Bergamo *et al*. 2024). The facilitation effect is further strengthened by the negative relationship between the abundance of conspecific flowers (and, therefore, plants with high floral display being more likely to self‐pollinate) and lower fruit set. Campos rupestres are characterized by harsh environmental conditions and varying pollination rates, in which plant–pollinator interactions are more likely to be opportunistic (Monteiro *et al*. 2021). In such environments, pollinators would exhibit generalist behaviour, leading to more pollinator sharing. In this context, selective pressures that foster the benefits of pollinator sharing through facilitation are expected (Eisen *et al*. 2020). Further studies using other species and more floral traits, such as resources offered by the flowers and flower colour, will be important to better understand the niche overlap between the plant species and the neighbourhood.

*Lantana hypoleuca* reproduction (likelihood of fruit production) peaked in plots with high heterospecific floral abundance, suggesting the benefits of sharing pollinators. Therefore, our study demonstrates the importance of considering the neighbourhood for plant reproduction. Moreover, the effect of the multiple variables describing the surrounding neighbourhood on *L. hypoleuca* depended on the pollination stage considered: pollen deposition and reproductive success. This stresses the complexity of indirect interactions between plant species sharing pollinators and the necessity of evaluating multiple reproductive stages of plant reproduction. Furthermore, as these context‐dependent relationships, such as plant richness and the abundance of flowering plants, can vary over the years, we emphasize the importance of long‐term studies to better understand the variation in neighbourhood composition and abundance. In summary, the main process influencing the reproduction of *L. hypoleuca* seems to be the facilitation provided by the shared pollinators with the neighbouring plant species.

## AUTHOR CONTRIBUTIONS

MDA, PJB, PKM and ARR conceived the ideas; MDA, JRB, APM collected the data; All authors contributed critically to the writing of the manuscript.

## CONFLICTS OF INTEREST STATEMENT

The authors declare no conflict of interest.

## Supporting information


**Table S1.** Visitation to *Lantana hypoleuca* flowers during the observation sessions. Plot is focal *L. hypoleuca*. Date is month/day of observation. Flower number is number of flowers in the focal *L. hypoleuca*. Visitor is identity of the visitor. NA represents absence of visitors.
**Table S2.** Matrix with abundance of plant species (Code) on each plot. Code is the same as in Table 1.
**Table S3.** Conspecific and heterospecific pollen found in each stigma and fruit and bugs of *L. hypoleuca*. Plot represents the focal *L. hypoleuca*.
**Table S4.** Dataset for the model to investigate the effect of community context on pollen deposition and on fruit set. Plot is the focal *L. hypoleuca*. CPD is conspecific pollen deposition. HPD and log(HPD) are heterospecific pollen deposition and its respective log transformation. CFA is conspecific floral abundance, and HFA is heterospecific floral abundance. Richness is plant species richness. Morphology is similarity in corolla length of *L. hypoleuca* in relation to other plant species.
