# Supplementary material for: Neighbourhood flower diversity increases reproductive success of Lantana hypoleuca Briq (Verbenaceae)
Source: Plant Biol (Stuttg). 2025 May 23;27(6):1158–64. doi: 10.1111/plb.70044 (PMC12477299; doi:10.1111/plb.70044)
Supplement: Supplementary file 1 — Table S1. Visitation to Lantana hypoleuca flowers during the observation sessions. Plot is focal L. hypoleuca. Date is month/day of observation. Flower number is number of flowers in the focal L. hypoleuca. Visitor is identity of the visitor. NA represents absence of visitors. Table S2. Matrix with abundance of plant species (Code) on each plot. Code is the same as in Table 1. Table S3. Conspecific and heterospecific pollen found in each stigma and fruit and bugs of L. hypoleuca. Plot represents the focal L. hypoleuca. Table S4. Dataset for the model to investigate the effect of community context on pollen deposition and on fruit set. Plot is the focal L. hypoleuca. CPD is conspecific pollen deposition. HPD and log(HPD) are heterospecific pollen deposition and its respective log transformation. CFA is conspecific floral abundance, and HFA is heterospecific floral abundance. Richness is plant species richness. Morphology is similarity in corolla length of L. hypoleuca in relation to other plant species. [file PLB-27-1158-s001.docx]

**Supplementary Material S1.** Visitation to *Lantana hypoleuca* flowers during the observation sessions. Plot is the focal *L. hypoleuca*. Date is the month/day of observation. Flower number is the number of flowers in the focal *L. hypoleuca*. Visitor is the identity of the visitor. NA represents the absence of visitors.

| **Plot** | **Date** | **Flower Number** | **Visitor** |
| --- | --- | --- | --- |
| P01 | 11/10 | 3 | NA |
|  | 11/17 | 2 | NA |
| P02 | 10/27 | 2 | NA |
| P03 | 10/27 | 37 | *Apis mellifera* |
|  | 11/05 | 14 | NA |
|  | 11/10 | 4 | NA |
| P04 | 10/27 | 13 | NA |
|  | 11/05 | 2 | Butterfly |
|  |  |  | Butterfly |
|  | 11/18 | 2 | NA |
| P05 | 10/27 | 48 | Bees |
|  |  |  | Bees |
|  |  |  | Bees |
|  |  |  | Bees |
|  |  |  | Bees |
|  |  |  | Bees |
|  | 11/17 | 47 | Bees |
|  |  |  | Butterfly |
|  |  |  | Bees |
|  | 11/18 | 43 | Bees |
| P07 | 10/25 | 4 | *Apis mellifera* |
|  | 11/18 | 3 | Bees |
|  | 11/19 | 3 | NA |
| P08 | 10/25 | 285 | *Apis mllifera* |
|  |  |  | Butterfly |
|  | 11/18 | 6 | NA |
|  | 11/19 | 5 | NA |
| P09 | 10/25 | 4 | NA |
|  | 11/18 | 4 | NA |
|  | 11/19 | 2 | NA |
| P10 | 10/27 | 95 | *Apis mellifera* |
|  |  |  | *Xylocopa* sp |
|  |  |  | *Apis mellifera* |
|  | 11/19 | 5 | NA |
| P11 | 10/25 | 200 | Bees |
|  |  |  | *Apis mellifera* |
|  |  |  | *Apis mellifera* |
|  | 11/18 | 22 | Butterfly |
|  | 11/19 | 14 | NA |
| P12 | 10/25 | 4 | NA |
|  | 11/03 | 2 | NA |
|  | 11/11 | 2 | NA |
| P13 | 10/25 | 18 | Butterfly |
|  |  |  | *Apis mellifera* |
|  | 11/03 | 15 | *Apis mellifera* |
|  | 11/03 | 1 | *Apis mellifera* |
|  | 11/18 | 4 | NA |
| P15 | 10/27 | 63 | *Apis mellifera* |
|  | 11/05 | 32 | Bees |
|  |  |  | Bees |
|  | 11/17 | 16 | Butterfly |
|  |  |  | Bees |
| P16 | 10/27 | 4 | NA |
|  | 11/05 | 9 | NA |
| P17 | 10/27 | 152 | *Trigona* sp |
|  |  |  | *Apis mellifera* |
|  |  |  | *Apis mellifera* |
|  |  |  | *Apis mellifera* |
|  |  |  | *Apis mellifera* |
|  |  |  | *Apis mellifera* |
|  |  |  | Butterfly |
|  | 11/03 | 94 | *Apis mellifera* |
|  |  |  | *Apis mellifera* |
|  |  |  | Butterfly |
|  | 11/17 | 8 | NA |
| P18 | 10/27 | 12 | Bees |
|  | 10/27 | 12 | Bees |
|  |  |  | Bees |
|  |  |  | Bees |
|  |  |  | Bees |
|  | 11/18 | 3 | *Bombus* sp |
|  | 11/19 | 2 | Butterfly |
| P19 | 10/27 | 420 | *Xylocopa* sp |
|  |  |  | *Apis mellifera* |
|  |  |  | *Apis mellifera* |
|  |  |  | *Apis mellifera* |
|  | 11/18 | 13 | NA |
|  | 11/19 | 15 | Bees |
| P20 | 10/27 | 31 | NA |
|  | 11/18 | 12 | Butterfly |
|  |  |  | Bees |
|  | 11/19 | 11 | Wasp |
| P21 | 10/26 | 86 | NA |
|  |  |  | Bees |
|  | 11/10 | 101 | Butterfly |
|  |  |  | Butterfly |
|  |  |  | Bees |
|  | 11/17 | 41 | *Apis mellifera* |
|  |  |  | Bees |
| P22 | 10/27 | 126 | *Apis mellifera* |
|  |  |  | *Apis mellifera* |
|  |  |  | *Apis mellifera* |
|  |  |  | Bees |
|  |  |  | Wasp |
|  |  |  | *Apis mellifera* |
|  | 11/18 | 11 | NA |
|  | 11/19 | 6 | NA |

Supplementary Material S2: Matrix with the abundance of plant species (Code) on each plot. Code is the same as code of Table 1.

| Code | P01 | P02 | P03 | P04 | P05 | P07 | P08 | P09 | P10 | P11 | P12 | P13 | P15 | P16 | P17 | P18 | P19 | P20 | P21 | P22 |
| --- | --- | --- | --- | --- | --- | --- | --- | --- | --- | --- | --- | --- | --- | --- | --- | --- | --- | --- | --- | --- |
| Ast_01 | 0 | 0 | 16 | 0 | 0 | 0 | 0 | 0 | 0 | 0 | 0 | 0 | 1 | 0 | 0 | 0 | 0 | 5 | 0 | 0 |
| Ast_03 | 0 | 0 | 0 | 0 | 0 | 0 | 0 | 0 | 0 | 0 | 0 | 0 | 0 | 3 | 0 | 0 | 0 | 0 | 0 | 0 |
| Ast_04 | 0 | 0 | 0 | 0 | 0 | 0 | 0 | 0 | 0 | 0 | 0 | 0 | 0 | 0 | 0 | 0 | 0 | 1 | 0 | 0 |
| Big_01 | 0 | 0 | 0 | 0 | 0 | 0 | 0 | 0 | 0 | 0 | 10 | 0 | 0 | 0 | 0 | 1 | 0 | 0 | 0 | 0 |
| Cha_01 | 0 | 0 | 0 | 0 | 0 | 0 | 0 | 0 | 0 | 0 | 0 | 0 | 0 | 0 | 0 | 0 | 0 | 0 | 5 | 0 |
| Cha_02 | 4 | 0 | 0 | 1 | 0 | 0 | 0 | 35 | 0 | 0 | 0 | 0 | 1 | 16 | 0 | 0 | 5 | 0 | 0 | 0 |
| Cup_01 | 0 | 0 | 0 | 0 | 0 | 0 | 3 | 0 | 0 | 0 | 0 | 0 | 2 | 0 | 0 | 0 | 0 | 0 | 0 | 0 |
| Cup_02 | 52 | 0 | 0 | 0 | 0 | 1 | 0 | 0 | 4 | 0 | 2 | 0 | 0 | 0 | 0 | 0 | 0 | 0 | 2 | 0 |
| Esp_01 | 0 | 0 | 4 | 0 | 15 | 22 | 4 | 0 | 0 | 2 | 0 | 0 | 0 | 0 | 0 | 0 | 4 | 2 | 5 | 2 |
| Esp_04 | 0 | 0 | 0 | 0 | 0 | 0 | 0 | 12 | 0 | 0 | 0 | 0 | 0 | 0 | 0 | 0 | 0 | 0 | 0 | 0 |
| Esp_07 | 0 | 0 | 0 | 0 | 0 | 0 | 16 | 0 | 0 | 0 | 0 | 0 | 0 | 0 | 0 | 0 | 0 | 0 | 0 | 0 |
| Esp_13 | 0 | 0 | 0 | 0 | 0 | 0 | 146 | 0 | 22 | 0 | 0 | 0 | 0 | 0 | 0 | 0 | 0 | 0 | 0 | 0 |
| Esp_16 | 0 | 0 | 0 | 0 | 0 | 0 | 3 | 0 | 0 | 18 | 0 | 0 | 0 | 0 | 0 | 0 | 0 | 0 | 0 | 0 |
| Esp_19 | 0 | 0 | 0 | 0 | 0 | 0 | 2 | 0 | 0 | 0 | 0 | 0 | 0 | 0 | 0 | 0 | 0 | 0 | 0 | 0 |
| Esp_21 | 0 | 0 | 0 | 17 | 0 | 0 | 0 | 0 | 0 | 0 | 0 | 0 | 0 | 0 | 0 | 0 | 0 | 0 | 0 | 0 |
| Esp_25 | 0 | 0 | 6 | 0 | 0 | 0 | 0 | 0 | 0 | 0 | 0 | 0 | 0 | 0 | 0 | 0 | 0 | 0 | 0 | 0 |
| Esp_26 | 6 | 0 | 0 | 0 | 0 | 0 | 0 | 0 | 0 | 0 | 0 | 0 | 0 | 0 | 0 | 0 | 0 | 0 | 9 | 0 |
| Esp_28 | 0 | 0 | 0 | 0 | 0 | 0 | 0 | 0 | 0 | 0 | 0 | 0 | 0 | 0 | 0 | 0 | 0 | 0 | 4 | 0 |
| Esp_29 | 0 | 3 | 0 | 0 | 0 | 0 | 0 | 0 | 0 | 0 | 0 | 0 | 0 | 0 | 0 | 0 | 0 | 0 | 0 | 0 |
| Esp_30 | 0 | 0 | 0 | 0 | 0 | 0 | 0 | 0 | 0 | 0 | 0 | 0 | 0 | 0 | 0 | 1 | 0 | 0 | 0 | 0 |
| Fab_01 | 0 | 0 | 0 | 0 | 34 | 0 | 3 | 0 | 0 | 0 | 0 | 0 | 2 | 0 | 0 | 0 | 0 | 0 | 0 | 0 |
| Lan_hy | 33 | 6 | 55 | 17 | 138 | 183 | 368 | 10 | 250 | 316 | 49 | 232 | 256 | 36 | 465 | 28 | 594 | 186 | 260 | 143 |
| Mal_01 | 0 | 0 | 7 | 0 | 0 | 0 | 0 | 0 | 0 | 0 | 0 | 0 | 1 | 0 | 7 | 4 | 0 | 0 | 2 | 0 |
| Mal_02 | 8 | 0 | 2 | 0 | 0 | 0 | 0 | 0 | 4 | 0 | 2 | 0 | 1 | 0 | 3 | 1 | 0 | 0 | 0 | 0 |
| Mel_01 | 0 | 0 | 0 | 0 | 0 | 0 | 0 | 0 | 0 | 0 | 0 | 0 | 0 | 0 | 0 | 0 | 1 | 5 | 0 | 0 |
| Mel_02 | 0 | 0 | 2 | 0 | 0 | 0 | 8 | 0 | 0 | 0 | 0 | 19 | 0 | 4 | 0 | 11 | 17 | 8 | 6 | 0 |
| Mel_05 | 0 | 0 | 0 | 0 | 0 | 0 | 0 | 0 | 0 | 0 | 0 | 0 | 0 | 0 | 0 | 0 | 0 | 0 | 1 | 0 |
| Pip_im | 0 | 0 | 0 | 0 | 0 | 0 | 0 | 0 | 0 | 0 | 1 | 0 | 0 | 0 | 8 | 0 | 0 | 0 | 0 | 0 |
| Vel_01 | 0 | 0 | 0 | 0 | 0 | 0 | 0 | 0 | 0 | 0 | 0 | 0 | 0 | 0 | 0 | 0 | 0 | 0 | 14 | 2 |

**Supplementary Material S3.** Conspecific and heterospecific pollen found in each stigma and fruit and bugs of L. hypoleuca. Plot represents the focal L. hypoleuca.

|  | Pollen | | Fruit | | Buds |
| --- | --- | --- | --- | --- | --- |
|  | Conspecific | Heterospecific | |  |  |
| P01 | 139 | 94 | | 12 | 18 |
|  | 153 | 42 | |  |  |
| P02 | 23 | 0 | | 3 | 15 |
|  | 129 | 21 | |  |  |
| P03 | 92 | 35 | | 6 | 8 |
|  | 27 | 32 | | 8 | 13 |
|  | 30 | 58 | |  |  |
| P04 | 106 | 74 | | 15 | 17 |
|  | 137 | 5 | | 9 | 28 |
| P05 | 76 | 44 | | 5 | 11 |
|  | 199 | 40 | | 12 | 13 |
| P07 | 63 | 9 | | 1 | 8 |
|  | 29 | 4 | |  |  |
| P08 | 24 | 9 | | 4 | 14 |
|  |  |  | | 3 | 10 |
| P09 | 67 | 0 | | 8 | 12 |
|  | 105 | 8 | |  |  |
| P10 | 109 | 52 | | 6 | 12 |
|  | 19 | 0 | |  |  |
| P11 | 28 | 4 | | 11 | 22 |
|  | 59 | 0 | |  |  |
| P12 | 24 | 0 | | 5 | 15 |
|  | 92 | 26 | |  |  |
| P13 | 37 | 9 | | 12 | 19 |
|  | 54 | 14 | |  |  |
| P15 | 54 | 22 | | 4 | 13 |
|  | 185 | 146 | |  |  |
| P16 | 58 | 7 | | 9 | 15 |
|  | 11 | 0 | |  |  |
| P17 | 123 | 23 | | 12 | 18 |
|  | 76 | 0 | |  |  |
| P18 | 31 | 24 | | 8 | 12 |
|  | 76 | 11 | |  |  |
| P19 | 8 | 0 | | 4 | 18 |
|  | 82 | 8 | | 2 | 8 |
|  | 33 | 0 | |  |  |
| P20 | 98 | 13 | | 7 | 10 |
|  | 123 | 27 | |  |  |
| P21 | 59 | 35 | | 17 | 22 |
|  | 68 | 6 | |  |  |
| P22 | 26 | 0 | | 3 | 14 |
|  | 80 | 13 | | 8 | 9 |
|  |  |  | | 19 | 28 |

**Supplementary Material S4.** Data set of the model to investigate the effect of community context on pollen deposition and on fruit set. Plot is the focal *L. hypoleuca*. CPD is conspecific pollen deposition. HPD and log(HPD) are heterospecific pollen deposition and its respective log transformation. CFA is conspecific floral abundance and HFA is heterospecific floral abundance. Richness is the plant species richness. Morphology is the similarity in corolla length of *L. hypoleuca* in relation to other plant species.

|  | Fruit | Buds | CPD | HPD | log(HPD) | CFA | HFA | Richness | Morphology |
| --- | --- | --- | --- | --- | --- | --- | --- | --- | --- |
| P01 | 12 | 18 | 146.00 | 68.00 | 4.22 | 16.50 | 35.00 | 4.50 | 0.34 |
| P02 | 3 | 15 | 76.00 | 10.50 | 2.35 | 6.00 | 3.00 | 2.00 | 0.00 |
| P03 | 14 | 21 | 49.67 | 41.67 | 3.73 | 18.33 | 12.33 | 3.00 | 0.61 |
| P04 | 24 | 45 | 121.50 | 39.50 | 3.68 | 5.67 | 6.00 | 1.67 | -1.33 |
| P05 | 17 | 24 | 137.50 | 42.00 | 3.74 | 46.00 | 16.33 | 3.00 | 0.50 |
| P07 | 1 | 8 | 46.00 | 6.50 | 1.87 | 61.00 | 7.67 | 1.67 | 0.73 |
| P08 | 7 | 24 | 24.00 | 9.00 | 2.20 | 122.67 | 61.67 | 3.67 | 0.66 |
| P09 | 8 | 12 | 86.00 | 4.00 | 1.39 | 3.33 | 15.67 | 2.33 | 0.47 |
| P10 | 6 | 12 | 64.00 | 26.00 | 3.26 | 125.00 | 15.00 | 2.50 | 0.86 |
| P11 | 11 | 22 | 43.50 | 2.00 | 0.69 | 105.33 | 6.67 | 1.67 | 0.59 |
| P12 | 5 | 15 | 58.00 | 13.00 | 2.56 | 16.33 | 5.00 | 3.00 | -0.33 |
| P13 | 12 | 19 | 45.50 | 11.50 | 2.44 | 77.33 | 6.33 | 2.00 | 0.66 |
| P15 | 4 | 13 | 119.50 | 84.00 | 4.43 | 85.33 | 2.67 | 3.00 | 0.58 |
| P16 | 9 | 15 | 34.50 | 3.50 | 1.25 | 18.00 | 11.50 | 3.00 | -1.05 |
| P17 | 12 | 18 | 99.50 | 11.50 | 2.44 | 155.00 | 6.00 | 3.00 | 0.61 |
| P18 | 8 | 12 | 53.50 | 17.50 | 2.86 | 9.33 | 6.00 | 2.67 | -0.29 |
| P19 | 6 | 26 | 41.00 | 2.67 | 0.98 | 198.00 | 9.00 | 2.67 | -0.17 |
| P20 | 7 | 10 | 110.50 | 20.00 | 3.00 | 62.00 | 7.00 | 2.67 | 0.09 |
| P21 | 17 | 22 | 63.50 | 20.50 | 3.02 | 86.67 | 16.00 | 4.33 | 0.18 |
| P22 | 30 | 51 | 53.00 | 6.50 | 1.87 | 47.67 | 1.33 | 2.33 | 0.50 |
